# High intratumoral expression of vimentin predicts histological transformation in patients with follicular lymphoma

**DOI:** 10.1038/s41408-019-0197-5

**Published:** 2019-03-18

**Authors:** Charlotte Madsen, Kristina Lystlund Lauridsen, Trine Lindhardt Plesner, Ida Monrad, Bent Honoré, Stephen Hamilton-Dutoit, Francesco d’Amore, Maja Ludvigsen

**Affiliations:** 1 Department of Hematology, Aarhus University Hospital, Aarhus, Denmark; 2 Institute of Pathology, Aarhus University Hospital, Aarhus, Denmark; 3 Department of Pathology, Copenhagen University Hospital, Copenhagen, Denmark; 4 Department of Biomedicine, Aarhus University, Aarhus, Denmark; 5 Department of Clinical Medicine, Aarhus University, Aarhus, Denmark

In western countries, follicular lymphoma (FL) is the second most common lymphoid malignancy comprising approximately 20% of all lymphomas, only exceeded by the other germinal center cell derived subtype diffuse large B-cell lymphoma (DLBCL). FL is a germinal center B-cell derived malignancy and histological transformation (HT) in FL patients, usually to DLBCL, is characterized by the replacement of the follicular growth pattern gradually substituted by invasion of diffusely invasive centroblasts. HT occurs in 10–70% of all patients with FL (presumably 20–40% of FL patients with grades 1–2) depending on HT definitions and follow-up criteria. This corresponds approximately to a 2% transformation rate per year^1–4^. The majority of untransformed FL follows an indolent clinical course with a median overall survival (OS) of 7–12 years^5–7^. In contrast, HT in FL patients is usually associated with a more progressive clinical course with adverse outcome compared to those FL patients without subsequent transformation^4,7,8^. The biological mechanism behind the transformation process from FL to DLBCL is still widely unclear. Although, it has been suggested that all FL will eventually develop into a more aggressive lymphoproliferation, other authors hold that transformation may not necessarily represent the final event in the natural history of FL, and that there may be a subgroup of patients in whom HT does not occur^1–3,9^. The mechanism responsible for HT is as yet unresolved, although this is a subject of great interest and has been examined in several previous studies. Various predictors have been suggested to affect the development of HT, e.g. FLIPI (age, hemoglobin level, nodal involvement, LDH level, and Ann Arbor stage), immunoglobulins, as well as treatment background, e.g. rituximab maintenance^10^. Previously, we reported the results of a mass spectrometry-based proteomic study that identified vimentin levels as a potential predictive marker^11^. Vimentin is a ubiquitously expressed major member of the intermediate filament protein family. It is strongly expressed in a broad range of mesenchymal cells, and in more limited subsets of normal epithelial cells. Vimentin is widely used as a routine IHC biomarker for phenotyping neoplasia of mesenchymal and melanocytic origin, and is also expressed in a smaller range of epithelial tumors. Current evidence suggests a correlation between vimentin overexpression and accelerated tumor growth and invasion in addition to its established association with the appearance of tissue changes known as epithelial–mesenchymal transition (EMT)^12^. However, the pathogenetic relationship of vimentin in the underlying molecular events mediating and promoting cancer progression remains unknown. Based on our previous findings from the proteomic study, we have in the present study investigated vimentin expression by immunohistochemistry in a larger cohort of pre-therapeutic diagnostic formalin-fixed paraffin embedded (FFPE) tumor tissue samples from (i) FL patients, grades 1–3A, without subsequent HT (non-transformed, nt-FL, *n* = 52) and (ii) FL patients with subsequent HT (sequential, s-FL, *n* = 43). For the s-FL patients, vimentin expression was assessed in both the pre-therapeutic tumor biopsy taken at FL diagnosis and the biopsy obtained at time of HT either DLBCL or FL grade 3B (s-tFL, *n* = 43).

Demographics and clinico-pathological features of the nt-FL and s-FL patients are listed in Table 1. The entire cohort consisted of 95 patients, including 51 males and 44 females. The median age was 56 years (range 25–83). Within this cohort, s-FL patients had a higher risk profile with more advanced clinical stage, higher FLIPI score, and higher tumor burden compared with nt-FL patients (Table 1). In s-FL, the median time to transformation was 4.8 years (range 0.5–21.4 years). The follow-up time for nt-FL was at least 10 years (Supplementary Figure S1-A). OS and PFS were significantly lower for s-FL compared with nt-FL with 5-years OS of 83 and 88% (*p* = 0.001) and 5-years PFS of 62 and 32% (*p* < 0.001), respectively. This is in accordance with data from comparable previously published cohorts (supplementary Figure S1-B)^1–3,5^. In addition, an adverse effect on survival is clearly seen in s-tFL at the time of HT (supplementary Figure S1-B). Expression levels of vimentin in the tumor tissue samples were significantly higher in s-FL compared with nt-FL (*p* < 0.001; Fig. 1a, b). Among s-FL patients, high levels of intratumoral vimentin expression were associated with significantly shorter transformation-free survival (TFS) compared with patients whose tumors has low vimentin expression (*p* = 0.020; Fig. 1c). This is also reflected in event-free survival (EFS), where transformation is an event (*p* = 0.023), but not in overall survival (OS) probably due to the long follow-up of up to 20 years in which time period patients may succumbed to unrelated mortality (*p* = 0.195), Fig. 1c. IHC evaluation identified different staining patterns for vimentin and for the pan-B-cell marker Pax-5 (Fig. 1a). Pax-5 showed nuclear staining of B-cells, and was, as expected, highly expressed in neoplastic B-cells. In contrast, vimentin showed a more widespread diffuse staining pattern throughout different cell types within the neoplastic tissue lesion. As the IHC stains were performed on consecutive sections, alignment of these allowed comparative visualization, virtually down to the single-cell level. This showed that only a fraction of B-cells (Pax-5 positive) were also positive for vimentin. Despite of the different staining patterns for Pax-5 and vimentin, both vimentin and Pax5 positive B-cells showed a significantly higher expression in s-FL compared with nt-FL (*p* < 0.001; Fig. 1b). Furthermore, a decrease in vimentin expression was observed when comparing s-FL with s-tFL (*p* < 0.001; Fig. 1a, b). This was not significant for Pax-5 (*p* = 0.306). The s-FL and s-tFL specimens analyzed in this study are sequential biopsies pairs from each included FL patient, who later developed HT. In most biopsy pairs, vimentin expression tended to decrease in the s-tFL specimen as compared with the s-FL biopsy (Supplementary Figure S2-A). However, there were a few exceptions where this pattern was not consistently present (Supplementary Figure S2-A). Pax-5 expression showed no tendency to decrease or increase in the analyzed sequential biopsies (Supplementary Figure S2-B). When applying a correlation analysis adjusted for sex, age, Ann Arbor stage, FLIPI, tumor burden, and LDH elevation, significant correlation between HT and of vimentin expression (*ρ* = 0.58, *p* < 0.001) as well as between HT and of Pax-5 expression (*ρ* = 0.54, *p* < 0.001) were found. No association with OS or PFS was seen for either vimentin or Pax-5 expression in nt-FL, s-FL, or s-tFL; similarly, no correlation with TFS in s-FL patients was observed (Supplementary Figure S3-A and S3-B).

**Table 1:** Clinico-pathological features

	All	nt-FL	s-FL	
	*n* = 95	*n* = 52	*n* = 43	
Characteristic	*n* (%)	*n* (%)	*n* (%)	*P*-value
Sex				
Male	51 (54)	24 (46)	27 (63)	NS
Female	44 (46)	28 (54)	16 (37)	
Age at FL diagnosis, years				
Median	56	55	57	NS
Range	25–83	35–83	25–78	
Ann Arbor stage				
I–II	31 (33)	26 (50)	7 (15)	<0.001
III–IV	62 (67)	26 (50)	36 (85)	
FLIPI				
Low	34 (37)	26 (52)	8 (20)	<0.001
Intermediate	31 (34)	18 (36)	13 (32)	
High	25 (29)	6 (12)	20 (49)	
*Missing*	4	2	2	
Nodal involvement ≥ 4	49 (53)	19 (37)	30 (73)	0.001
*Missing*	3	1	2	
B-symptoms	22 (24)	9 (18)	13 (32)	NS
*Missing*	5	2	3	
Performance score ≥ 2	2 (2)	2 (4)	0 (0)	NS
*Missing*	3	1	2	
Bone marrow involvement	28 (35)	10 (22)	18 (50)	0.011
*Missing*	14	7	7	
Anemia	6 (7)	1 (2)	5 (12)	NS
*Missing*	3	1	2	
LDH-elevation	16 (18)	4 (8)	12 (29)	0.012
*Missing*	4	2	2	
FL histology				
FL NOS	62(65)	37 (71)	25 (58)	NS
FL grade 1–2	29 (30)	13 (25)	16 (37)	
FL grade 3 A	4 (4)	2 (4)	2 (5)	
Initial treatment strategy (at FL)				
Alkylator-based, antracyclin void	26 (28)	17 (33)	9 (20)	NS
Antracyclin-based	9 (9)	2 (4)	7 (16)	
Chlorambucil	21 (22)	12 (23)	9 (21)	
Rituximab monotherapy	9 (9)	2 (4)	7 (16)	
Radiation only	6 (6)	4 (8)	2 (5)	
Wait-and-watch	21 (22)	14 (27)	7 (16)	
Other	3 (3)	1 (2)	2 (4)	
R-Chemo	21 (22)	9 (17)	12 (28)	
Vimentin expression level				
Low	49 (52)	36 (69)	13 (30)	<0.001
High	46 (48)	16 (31)	30 (70)	
Pax-5 expression level				
Low	56 (59)	43 (83)	13 (30)	<0.001
High	39 (41)	9 (17)	30 (70)	

*FL* follicular lymphoma, *LDH* lactate dehydrogenase, *NS* not significant, *nt-FL* non-transformed FL, *s-FL* sequential FL, *R-chemo* rituximab in combination with chemotherapy

**Fig. 1:**
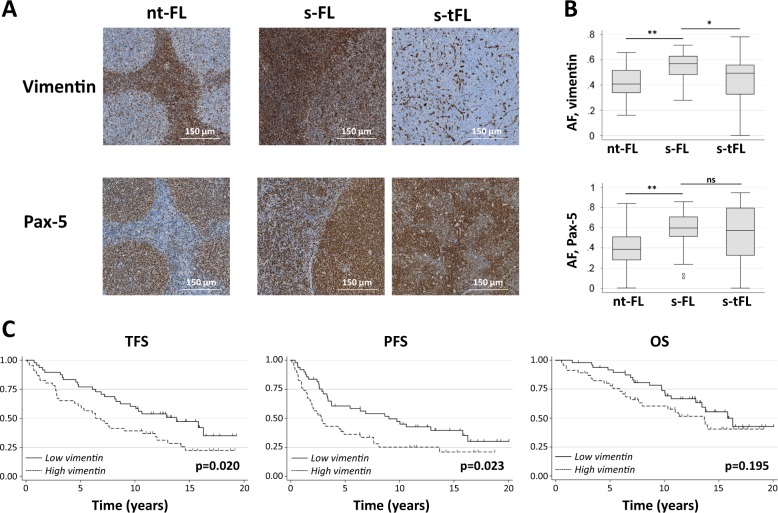
Vimentin expression. **a** Representative IHC stainings for vimentin and Pax-5 in nt-FL and corresponding s-FL/s-tFL. Stains were performed on consecutive slides. For each stain, the figures shown are from tissue samples from the same patients. Magnification 1:20. **b** Total expression of vimentin and Pax-5 by digital pathology as area fraction (AF) showing significant differences between nt-FL and s-FL (vimentin and Pax-5) and between s-FL and s-tFL (vimentin), *p < 0.01 and **p < 0.001, (Supplementary Patients and Methods). ns: not significant. **c** Transformation-free survival (TFS), progression-free survival (PFS), and overall survival (OS) in FL patients (ntFL and s-FL) shows an increased risk of transformation when FL presents with high vimentin expression. High/low vimentin was defined from the median of all FL patients (cut-off AF vimentin: 0.50088, Pax-5: 0.55291). AF, area fraction; FL, follicular lymphoma; ns, not significant, ntFL, non-transformed FL; OS, Overall survival, PFS, progression-free survival, s-FL, sequential FL; s-tFL, sequential transformed FL, TFS, transformation-free survival

To the best of our knowledge, this is the first study which has identified IHC vimentin expression as a possible predictive biomarker of HT in FL. We have used the 2016 WHO definition of transformation, i.e., in which FL grade 3B is included as a transformed histology. Due to our criteria requiring a minimum of 10 years’ follow-up, the present study cohort has a median age of 56 years, which is almost a decade lower than the median age (65 years) of a classical Caucasian FL population. Due to the follow-up length criteria, most of the patients were diagnosed at a time where upfront rituximab maintenance had not yet been introduced as an option in the management of FL. This raises the question of whether the impact of intratumoral vimentin expression would retain its predictive value for HT also in a patient cohort treated with rituximab maintenance.

Previously large-scale studies have reported differential expression of vimentin at either the genomic, RNA or protein level in transformed versus non-transformed FL samples^11,13,14^. Our own previous mass spectrometry based proteomic study performed on a size limited cohort of 12 patients, showed a vimentin fragment with a 3-fold significant higher expression levels in pre-therapeutic tumor tissue samples from nt-FL patients compared with s-FL patients^11^. Identification of this fragment was obtained on the basis of 9 protein peptides. In FL, the tumor lesions are composed of an admixture of tumor cells (centrocytic and centroblastic B-cells) and tumor microenvironmental by-standers such as benign lymphocytes, macrophages, follicular dendritic cells, stromal cells etc. This suggests that the expression level of vimentin could be a predictive marker of HT in FL patients at the time of initial diagnosis. In the present study, we have focused on evaluating vimentin in a larger cohort with a standard-in-use antibody and routine IHC methodology. Interestingly, a previous report from Cha et al. found that full-length vimentin, but not *N*-truncated fragments of the protein, was recognized by 42 out of 217 tested FL immunoglobulins (Igs) as a shared autoantigen. The epitope was located in the *N*-terminal region of the protein (between amino acid residues 224 and 259) in all vimentin-reactive tumor Igs^15^. This recognition of vimentin as a shared autoantigen by non-stereotyped FL B-cell receptors suggests a role of this protein in promoting malignant transformation and disease progression. In addition, another report also identified vimentin expression as a mediator of a drug-resistant invasive phenotype in diffuse large B-cell lymphoma^16^. In the light of these previous reports and the results of the present study, further investigations on a possible functional role of vimentin in the histological transformation process of FL is warranted.

In conclusion, this is the first study to identify vimentin as a possible predictive biomarker of subsequent HT in FL patients. This biomarker can be assessed at the time of FL diagnosis by a standard histopathological method that is widely available in diagnostic pathology laboratories. Our findings warrant validation in a larger independent cohort also including patients exposed for rituximab maintenance. If confirmed, this finding would support the use of intratumoral vimentin expression as a useful parameter for early risk-adapted management of FL patients.

## Supplementary information

Supplementary Patients and Methods

Supplementary Figure S1.

Supplementary Figure S2.

Supplementary Figure S3.

Supplementary Figure legends.
