# Supplementary material for: High intratumoral expression of vimentin predicts histological transformation in patients with follicular lymphoma
Source: Blood Cancer J. 2019 Mar 18;9(4):35. doi: 10.1038/s41408-019-0197-5 (PMC6423140; doi:10.1038/s41408-019-0197-5)
Supplement: Supplementary file 1 — Supplementary Patients and Methods [file 41408_2019_197_MOESM1_ESM.docx]

**Supplementary Patient and Methods**

The present study was approved by the Regional Research Ethics Committee (1-10-72-276-13) and by the Danish Data Protection Agency (2013-41-1867).

*Patients*

All patients diagnosed with FL at the Department of Hematology in Aarhus, in the period 1990-2015, were identified through the Danish Pathology Registry and the Danish National Lymphoma Registry (LYFO) (1–3). Pathology reports of all diagnostic biopsies for patients diagnosed with FL were reviewed (n=617). Patients were categorized as FL ‘non-transformed’ (nt-FL, n=52) if they had a follow-up period of at least 10 years without sign of transformation, *i.e.* alive and without a secondary histologically verified diagnosis of DLBCL or FL grade 3B. Patients with HT were identified and categorized as sequentially transformed FL (s-FL/s-tFL, n=43), if they had a primary diagnosis of FL grade 1-3A (s-FL) and subsequently, *i.e.* after at least 6 months, a biopsy-proven diagnosis of DLBCL or FL grade 3B (s-tFL). The median time to transformation (TTT) was 4.8 yrs (range 0.5-21.4 years). Patients were included if sufficient FFPE tumor tissue was available, collected at the time of the initial FL diagnosis, either from an excision biopsy or a core needle biopsy were available. Additionally, FFPE tissue samples from the diagnostic biopsy at the time of HT were retrieved. Only patients with a complete pair of biopsies at s-FL and s-tFL diagnoses were included. All tumor biopsies were reviewed and reclassified by an experienced hematopathologist (TP) according to the 2008 WHO Classification of Tumors of the Haematopoietic and Lymphoid Tissues to validate the diagnosis of FL and tFL (4,5). As recommended and applied in several previous reports, the diagnosis of HT in the present study is based on histological evaluation (6–13). Other, more clinical, criteria have been used in studies of transformation in FL. These criteria have included clinical parameters such as rise in LDH, rapid localized nodal growth, new extra nodal sites of disease, presence of B‐symptoms, and/or hypercalcemia (6,13–15). Data on demographics, clinico-pathological features and treatment was acquired by cross linking with the Danish Lymphoma Registry (LYFO) and, if needed for data-completeness, with patient records. Only one patient in the cohort received maintenance treatment with rituximab.

*Immunohistochemistry*

Immunohistochemical staining was performed on 4 μm paraffin-embedded tissue sections. Pax-5 (rabbit monoclonal antibody SP34, catalogue no. 790-4020, Ventana Medical Systems, Tucson, AZ) immunohistochemical (IHC) stainings was used to identify B-cells. IHC stains for Pax-5 and vimentin (mouse monoclonal antibody V9, catalogue no 790-2917, Ventana) were performed on consecutive sections and stained on the Ventana Benchmark Ultra automated staining system (Ventanax). For primary antibody detection the Ventana Optiview™ DAB detection kit (Ventana) was used. Appendix, tonsil, liver, and pancreas section were used as positive/negative tissue controls.

*Digital pathology*

Images of stained tissue slides were captured and digitalized at a magnification of x20 using the Hamamatsu Nanozoomer 2.0HT scanner (Hamamatsu Photonics, Hamamatsu City, Japan). Expression levels of Pax-5 and Vimentin were digitally quantified using VIS (Visiopharm Integrator System 2018.4, Visiopharm, Hørsholm, Denmark) as previously described (16,17). An area fraction (AF) of IHC positivity (*i.e* including both tumor and non-tumor cells together) was computed as the IHC stained area normalized to the total tissue area in the biopsy, *i.e*. region of interest (ROI). This was designated as the IHC protein expression score for each patient sample. All stained samples were manually reviewed and the total ROI was adjusted to exclude technical confounding factors such as folds, staining artefacts, tissue damage. In addition, distinct areas of non-lymphoid tissue, such as fatty tissue and necrosis were discarded. Protein expression was designated as the area fraction of either vimentin or Pax-5 stains.

*Statistical analyses*

Differences between mean area fractions of vimentin and Pax-5 were calculated using Student’s t-test or ANOVA for repeated measures after normality was tested. Patient characteristics of categorical and continuous variables were tested using Fisher’s exact test, Student’s t-test or Wilcoxon rank-sum test as appropriate. Correlation analyses were done by Spearman’s rank correlation. OS was defined from the date of initial diagnosis of either FL or HT to the date of death by any cause or censoring; progression-free survival (PFS) was calculated from the date of the histological diagnosis of either FL or HT to the date of progression/relapse or censoring; transformation-free survival (TFS) was calculated from the date of initial FL diagnosis to the date of biopsy proven HT. All time related end-points were estimated by the Kaplan-Meier method and compared by the log-rank test. For survival analysis on digitally quantified specimens, two groups for each staining (vimentin and Pax-5) were based on the median area fraction, *i.e.* the 50^th^ percentile for high versus low intratumoural expression level as described (cut-off AF vimentin: 0.50088, Pax-5: 0.55291) (17). Significant differences were defined as p<0.05. All statistical analyses were performed using STATA IC 14 (StataCorp, College Station, TX).

**Supplement references**

1. Arboe B, El-Galaly TC, Clausen MR, Munksgaard PS, Stoltenberg D, Nygaard MK, et al. The Danish National Lymphoma Registry: Coverage and Data Quality. PLoS One. 2016;11(6):e0157999.

2. Arboe B, Josefsson P, J\orgensen J, Haaber J, Jensen P, Poulsen CB, et al. Danish National Lymphoma Registry. Clin Epidemiol. 2016;Volume 8:577–81.

3. Erichsen R, Lash TL, Hamilton-Dutoit SJ, Bjerregaard B, Vyberg M, Pedersen L. Existing data sources for clinical epidemiology: the Danish National Pathology Registry and Data Bank. Clin Epidemiol. 2010;2:51–6.

4. Swerdlow SH, Campo E, Harris NL, Jaffe ES, Pileri SA, Stein H, et al. WHO Classification of Tumours of Haematopoietic and Lymphoid Tissues. World Health Organization Calssification of Tumours of Haematopoietic and Lymphoid Tissue. 2008.

5. Swerdlow SH, Campo E, Pileri SA, Harris NL, Stein H, Siebert R, et al. The 2016 revision of the World Health Organization (WHO) classification of lymphoid neoplasms. Blood. 2016;

6. Al-Tourah AJ, Gill KK, Chhanabhai M, Hoskins PJ, Klasa RJ, Savage KJ, et al. Population-based analysis of incidence and outcome of transformed non-Hodgkin’s lymphoma. J Clin Oncol. 2008;

7. Montoto S, Davies AJ, Matthews J, Calaminici M, Norton AJ, Amess J, et al. Risk and clinical implications of transformation of follicular lymphoma to diffuse large B-cell lymphoma. J Clin Oncol. 2007 Jun;25(17):2426–33.

8. Cullen MH, Lister TA, Brearley RL, Shand WS, Stansfeld AG. Histological transformation of non‐hodgkin’s lymphoma. A prospective study. Cancer. 1979;

9. Acker B, Hoppe RT, Colby T V., Cox RS, Kaplan HS, Rosenberg SA. Histologic conversion in the non-Hodgkin’s lymphomas. J Clin Oncol. 1983;

10. Yuen AR, Kamel OW, Halpern J, Horning SJ. Long-term survival after histologic transformation of low-grade follicular lymphoma. J Clin Oncol. 1995;

11. Bastion Y, Sebban C, Berger F, Felman P, Salles G, Dumontet C, et al. Incidence, predictive factors, and outcome of lymphoma transformation in follicular lymphoma patients. J Clin Oncol. 1997 Apr;15(4):1587–94.

12. Montoto S, Fitzgibbon J. Transformation of indolent B-cell lymphomas. Journal of Clinical Oncology. 2011.

13. Ersbøll J, Schultz HB, Pedersen‐Bjergaard J, Nissen NI. Follicular low‐grade non‐Hodgkin’s lymphoma: Long‐term outcome with or without tumor progression. Eur J Haematol. 1989;

14. Horning SJ, Rosenberg SA. The Natural History of Initially Untreated Low-Grade Non-Hodgkin’s Lymphomas. N Engl J Med. 1984;

15. Giné E, Montoto S, Bosch F, Arenillas L, Mercadal S, Villamor N, et al. The Follicular Lymphoma International Prognostic Index (FLIPI) and the histological subtype are the most important factors to predict histological transformation in follicular lymphoma. Ann Oncol. 2006;

16. Pedersen MB, Danielsen A V., Hamilton-Dutoit SJ, Bendix K, Nørgaard P, Møller MB, et al. High intratumoral macrophage content is an adverse prognostic feature in anaplastic large cell lymphoma. Histopathology. 2014;

17. Vase M, Ludvigsen M, Bendix K, Dutoit SH, Hjortebjerg R, Petruskevicius I, et al. Predictive value of galectin-1 in the development and progression of HIV-associated lymphoma. AIDS. 2017.
