# Supplementary figures and images for: High intratumoral expression of vimentin predicts histological transformation in patients with follicular lymphoma
Source: Blood Cancer J. 2019 Mar 18;9(4):35. doi: 10.1038/s41408-019-0197-5 (PMC6423140; doi:10.1038/s41408-019-0197-5)

**Supplementary Figure S1**

**
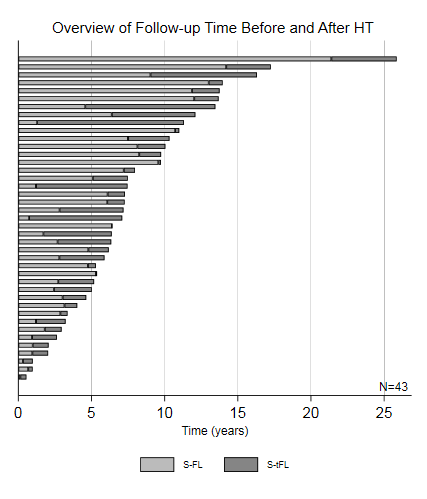
A**

**B**

**
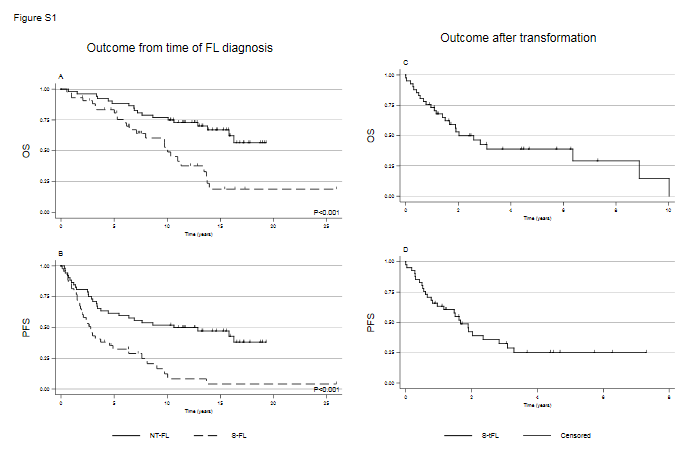
**

Supplement: Supplementary file 2 — Supplementary Figure S1. [file 41408_2019_197_MOESM2_ESM.docx]

**Supplementary Figure S2**

**A**

**
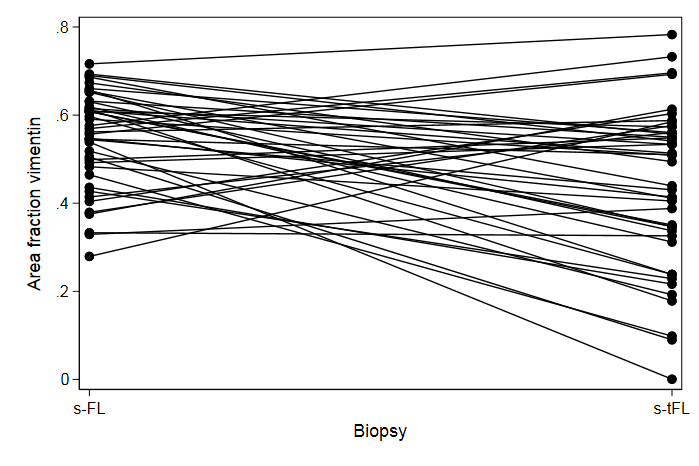
**

**B**

**
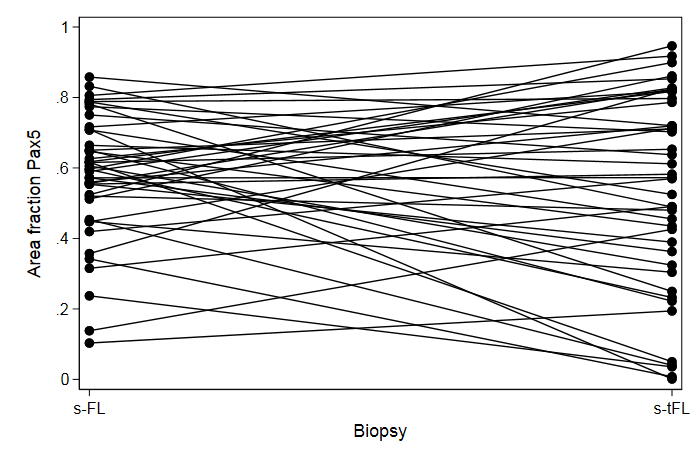
**

Supplement: Supplementary file 3 — Supplementary Figure S2. [file 41408_2019_197_MOESM3_ESM.docx]

**Supplementary Figure S3**

**A** Outcome by vimentin

**
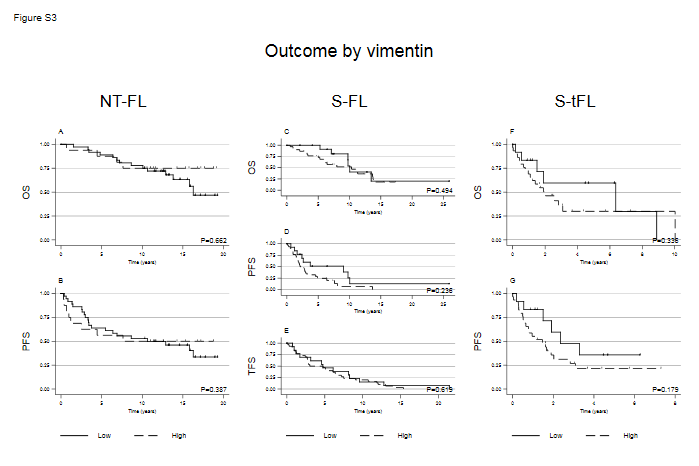
** nt-FL s-FL s-tFL

**B** Outcome by Pax-5

**
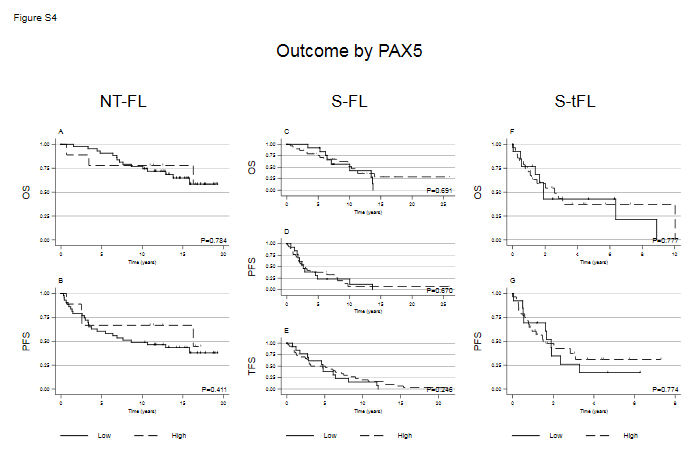
** nt-FL s-FL s-tFL

Supplement: Supplementary file 4 — Supplementary Figure S3. [file 41408_2019_197_MOESM4_ESM.docx]
