# Supplementary material for: High intratumoral expression of vimentin predicts histological transformation in patients with follicular lymphoma
Source: Blood Cancer J. 2019 Mar 18;9(4):35. doi: 10.1038/s41408-019-0197-5 (PMC6423140; doi:10.1038/s41408-019-0197-5)
Supplement: Supplementary file 5 — Supplementary Figure legends. [file 41408_2019_197_MOESM5_ESM.docx]

**Supplementary Figure legends**

Figure S1. **Cohort characteristics.** A. Swimmers plot of time to transformation. For s-FL patient the time to transformation was 4.8 years (range 0.5-21.4 years). B. Outcome analyses for nt-FL, s-FL and s-tFL. Outcome analyses, *i.e* overall survival (OS) and progression free-survival (PFS) of nt-FL and s-FL, show an adverse outcome for s-FL compared with nt-FL (a,b) as well as s-tFL (c,d)

Figure S2. **Corresponding vimentin and Pax-5 expression for s-FL and s-tFL pairs.** A. Vimentin expression in s-FL and s-tFL showed a significant difference in expression, However, no uniform pattern of decrease of vimentin expression in the transformation of s-FL to s-tFL was observed when specified for each patient. B. Pax-5 showed no difference in expression level in s-FL compared with s-tFL.

Figure S3. **Association of vimentin and Pax-5 expression levels on overall survival (OS), progression free survival (PFS) and transformation free survival (TFS).** A. Vimentin. No impact on outcome is observed for nt-FL (a,b), s-FL (c,d,e) and s-tFL (f,g) according to high and low (median AF) vimentin is observed. B. Pax-5. No impact on outcome for nt-FL (a,b), s-FL (c,d,e) and s-tFL (f,g) according to high and low (median area fraction) Pax-5 is observed.
